# Intra-abdominal etiology of post-abdominoplasty pain: report of 2 cases

**DOI:** 10.1080/23320885.2026.2678774

**Published:** 2026-06-02

**Authors:** Julia Ting, Diwakar Phuyal, Bahar Bassiri Gharb, Antonio Rampazzo

**Affiliations:** Dermatology and Plastic Surgery Institute, Cleveland Clinic, Cleveland, OH, USA

**Keywords:** Abdominoplasty, postoperative pain, small bowel obstruction, diagnostic delay, body contouring

## Abstract

Abdominoplasty is a commonly performed body-contouring procedure with a predictable postoperative course. Early abdominal pain is typically attributed to surgical site discomfort, edema, rectus plication, or common complications such as seroma or hematoma. However, intra-abdominal pathology may present with overlapping or nonspecific symptoms, increasing the risk of diagnostic delay. We report two cases of postoperative abdominal pain following abdominoplasty in which intra-abdominal etiologies were identified or strongly considered. In Case 1, a 71-year-old woman with prior Roux-en-Y gastric bypass developed progressive abdominal pain and obstructive symptoms on postoperative day 1. Imaging revealed small bowel obstruction, and diagnostic laparoscopy confirmed an internal hernia through a Petersen’s defect requiring surgical intervention. In Case 2, a 34-year-old woman presented with progressive right lower quadrant pain and swelling after a combined reduction mammaplasty and abdominoplasty. Imaging demonstrated an incidental ovarian hemorrhagic cyst, and she was managed conservatively with resolution of symptoms. In both cases, the abdominal wall and surgical sites were clinically benign. These cases highlight that postoperative abdominal pain after abdominoplasty is not always attributable to the abdominal wall or operative field. Overlapping symptoms may contribute to diagnostic anchoring and delayed recognition of intra-abdominal pathology. Maintaining a broad differential diagnosis, particularly in patients with prior abdominal surgery or atypical symptom progression, is essential. Prompt evaluation and multidisciplinary collaboration can reduce morbidity and prevent delays in care.

## Introduction

Abdominoplasty is a commonly performed body-contouring procedure that is generally associated with a predictable postoperative course [1,2]. Early postoperative abdominal pain is typically attributed to surgical site discomfort, soft tissue edema, rectus plication, or common complications such as seroma or hematoma [3,4]. As a result, postoperative symptoms are often managed expectantly, with reassurance and conservative measures [5].

However, abdominal pain following abdominoplasty is not always attributable to the abdominal wall or operative field. In rare cases, intra-abdominal pathology may present with nonspecific or atypical symptoms that overlap with expected postoperative findings, increasing the risk of diagnostic delay. Failure to recognize these alternative etiologies can result in prolonged morbidity and delayed intervention.

These 2 cases highlight the importance of maintaining a broad differential diagnosis when evaluating postoperative abdominal pain after abdominoplasty and underscores the need for timely evaluation of potential intra-abdominal causes to avoid delayed diagnosis and associated complications.

## Case report

Written informed consent was obtained from the patients for publication of this case report and accompanying images.

### Case 1

**Figure 1.**
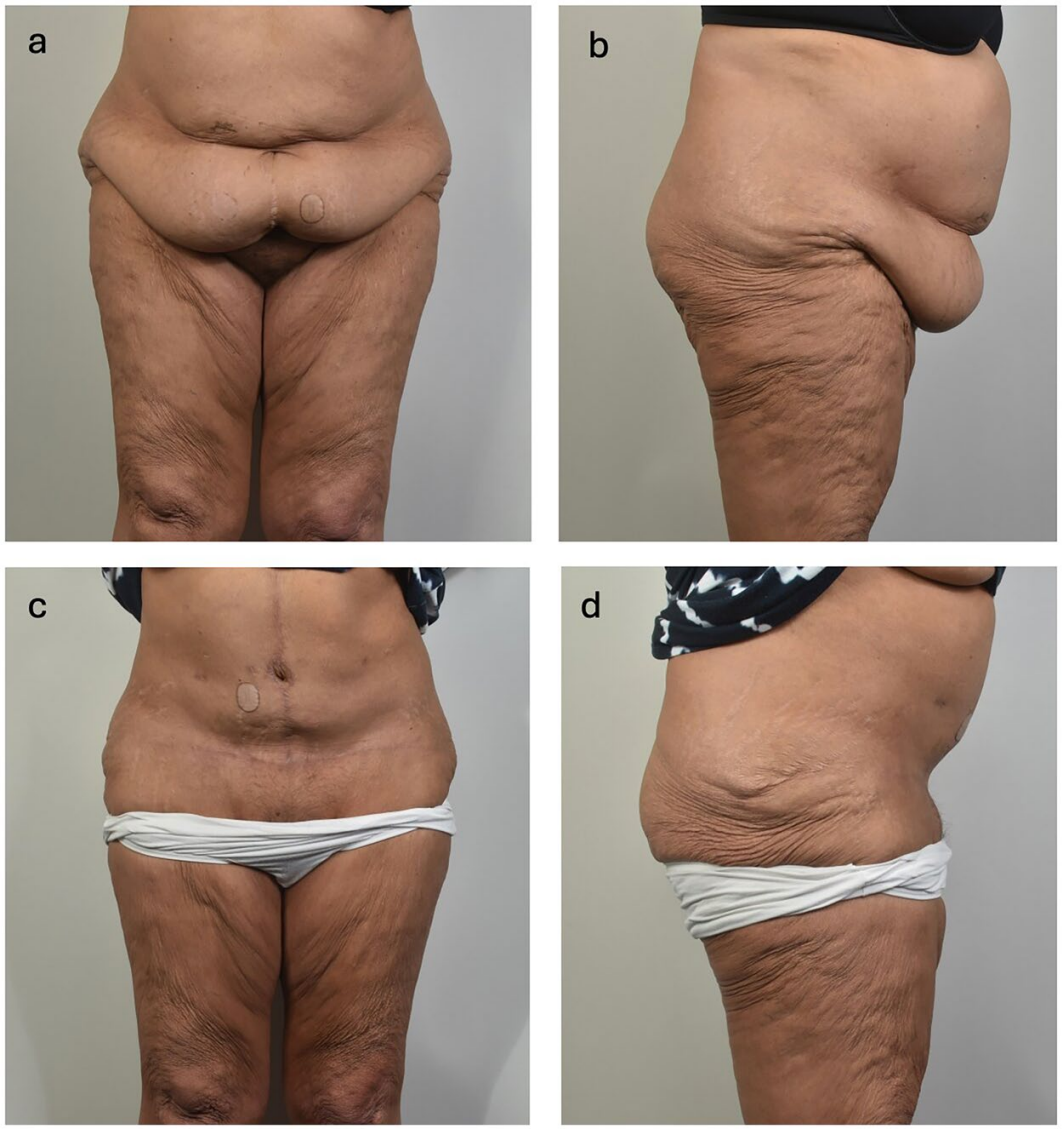
Preoperative (1a, 1b) and postoperative (1c, 1d) clinical photographs demonstrating abdominal contour before and after abdominoplasty. Anterior (left) and lateral (right) views are shown.

A 71-year-old woman (BMI 32.6) with a history of hypertension and prior bariatric surgery—sleeve gastrectomy followed by Roux-en-Y gastrojejunostomy in 2022—presented for abdominoplasty due to redundant abdominal skin associated with chronic back pain and recurrent intertrigo. She had lost 80 pounds following bariatric surgery and an additional 17 pounds after initiating semaglutide four months prior to surgery (Figure 1a, 1b). Preoperative computed tomography demonstrated no evidence of ventral hernia, rectus diastasis, bowel dilation, or other intra-abdominal pathology.

She underwent a fleur-de-lis abdominoplasty with a lower transverse and vertical incision. Large perforating vessels were ligated, and excess tissue was excised in a stepwise fashion with meticulous hemostasis. Perforating towel clamps were used to approximate the skin edges and prevent over-resection. A total of 2,200 grams of tissue was removed. The Scarpa fascia and skin were closed in layers, the umbilicus was exteriorized, and compression garments were applied. She was transferred to the recovery unit in stable condition.

On postoperative day (POD) 1, the Adult Medical Emergency Team was activated for severe hypertension (178/110 mmHg), chest pressure, and dyspnea, with oxygen saturation of 91% on room air. These symptoms were accompanied by nausea, emesis, and absence of flatus or bowel movements. Computed tomography imaging suggested a small bowel obstruction with a transition point near the jejunojejunostomy. An initial upper gastrointestinal study was unremarkable, and bowel function briefly returned; however, repeat imaging demonstrated progressive small bowel dilation consistent with a partial obstruction.

On POD 7, diagnostic laparoscopy revealed a dilated Roux limb, an omental band causing partial obstruction, stenosis proximal to the jejunojejunostomy, and an internal hernia of the biliopancreatic limb through a Petersen’s defect. She subsequently underwent an enteroenterostomy bypassing the prior jejunojejunostomy and closure of the Petersen’s defect.

Her hospital course was prolonged, totaling 13 days, and was further complicated by readmission for pneumonia and a urinary tract infection, both treated with levofloxacin. From a plastic surgery perspective, her postoperative course was otherwise uncomplicated, with no evidence of hematoma, seroma, or wound infection. Surgical drains were removed within three weeks. At five-month follow-up, she reported mild lower abdominal tightness and residual abdominal distension without associated gastrointestinal symptoms (Figure 1c, 1d).

### Case 2

**Figure 2.**
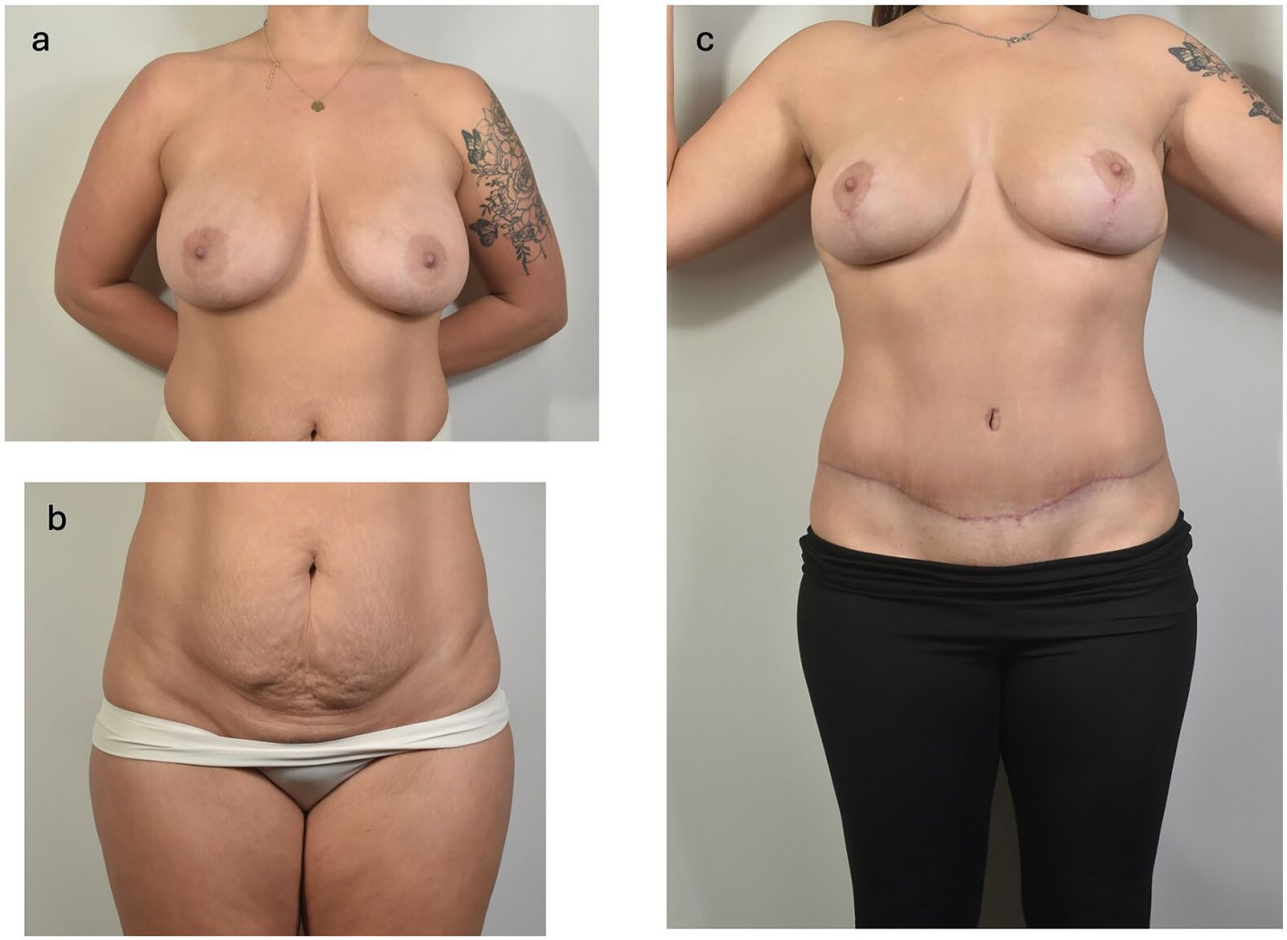
Preoperative (2a, 2b) and postoperative (2c) clinical photographs demonstrating breast and abdominal contour before and after bilateral reduction mammaplasty with abdominoplasty.

A 34-year-old woman (BMI 28.3) presented for evaluation of excess abdominal skin and symptomatic macromastia, reporting chronic upper and lower back pain and daily shoulder pain refractory to physical therapy and analgesics (Figure 2a, 2b). She underwent bilateral breast reduction using a superior pedicle with Wise-pattern skin excision, with 202 g removed from the right breast and 263 g from the left. This was followed by supraumbilical liposuction (350 cc aspirated using a 4-mm cannula) and lipo-abdominoplasty.

A lower abdominal incision was made, and dissection proceeded cephalad to the level of the umbilicus. After flap elevation, rectus fascial plication was performed. Excess skin was excised in a stepwise fashion using perforating towel clamps to avoid over-resection. Hemostasis was achieved, the umbilicus was exteriorized and secured with absorbable sutures, and two Blake drains were placed. Scarpa’s fascia and skin were closed in layers. The total resection weight was 966 g. She was discharged after an uneventful 28-hour hospital stay.

On POD 4, she returned with right-sided abdominal swelling, a pressure sensation, and mild erythema, without evidence of fluid collection or infection; she was counseled regarding signs and symptoms of infection. At follow-up on POD 8, both drains were removed. On POD 9, she presented to the emergency department with right lower quadrant pain and swelling extending to the groin and vaginal area, which she described as “a balloon inside my stomach” that feels like it might “pop” with certain movement. Imaging demonstrated expected postoperative changes and an incidental 3.9-cm right ovarian hemorrhagic cyst.

She was managed conservatively with activity restriction and stool softeners. Her pain, swelling, and erythema gradually resolved over several weeks. At 2.5-month follow-up, she was healing well, pain-free, satisfied with the surgical outcome, and reported only mild abdominal numbness and hypertrophic scarring (Figure 2c).

## Discussion

Abdominoplasty is a commonly performed procedure with a well-established safety profile, and postoperative abdominal pain is most often attributed to expected surgical site discomfort, seroma, hematoma, or musculoskeletal strain related to rectus plication. However, as demonstrated in these cases, abdominal pain following abdominoplasty may originate from intra-abdominal pathology, and anchoring on superficial or procedure-related explanations can delay diagnosis and appropriate management.

Patients undergoing abdominal contouring may present with nonspecific symptoms, including pain, swelling, abdominal pressure, or gastrointestinal complaints, which overlap with typical postoperative findings. This diagnostic ambiguity is compounded by the frequent use of combined procedures, postoperative narcotic use, and activity restrictions that may mask evolving intra-abdominal pathology. In such cases, a high index of suspicion is required, particularly when symptoms are progressive, disproportionate to the expected postoperative course, or associated with systemic or gastrointestinal features such as nausea, vomiting, distension, or obstipation. Additionally, rectus fascial plication is a common component of abdominoplasty which increases intra-abdominal pressure and may unmask previously asymptomatic intra-abdominal pathology. In patients with prior Roux-en-Y gastric bypass (Case 1), this may precipitate acute presentation of internal hernias, such as through a Petersen’s defect, and should be considered when evaluating postoperative symptoms [6,7].

This phenomenon is supported by prior reports of intra-abdominal complications following abdominoplasty in post-bariatric patients. Cases of early postoperative Petersen’s hernia have been described, with proposed mechanisms including increased intra-abdominal pressure from rectus plication and compression garments precipitating acute herniation in previously asymptomatic defects [8]. Additionally, abdominoplasty-associated increases in intra-abdominal pressure have been linked to exacerbation or recurrence of pre-existing hernias in this population [9]. These findings suggest that such complications are not merely coincidental but may reflect a physiologically mediated process.

The presented case underscores the importance of considering intra-abdominal etiologies in the differential diagnosis of postoperative abdominal pain, even when imaging demonstrates expected postoperative changes and the surgical site appears benign. Early reassurance based solely on the absence of wound complications may contribute to delayed recognition of serious underlying pathology. For example, in Case 1, although symptoms began on POD 1, operative intervention was not performed until POD7. This delay likely reflects the diagnostic challenges inherent to the postoperative setting, where symptoms overlap with expected recovery and imaging findings may be initially inconclusive. Anchoring bias, or prematurely attributing symptoms to the surgical site or expected postoperative course, may further contribute to delayed escalation of care [10,11]. Timely cross-sectional imaging and multidisciplinary collaboration are critical when symptoms persist or evolve, especially in patients with prior abdominal or pelvic pathology, prior surgeries, or atypical pain patterns. This is particularly relevant when considering non-surgical intra-abdominal etiologies that may further confound the postoperative presentation. In Case 2, no clear pathophysiological relationship between abdominoplasty and the development of a hemorrhagic ovarian cyst was identified. Although increased intra-abdominal pressure or compression may be hypothesized, this case more likely represents a diagnostic challenge, with gynecologic pathology mimicking expected postoperative symptoms.

Delayed diagnosis of intra-abdominal complications can lead to prolonged hospitalizations, increased morbidity, and patient distress. While conservative management may be appropriate in select cases, close clinical follow-up and reassessment are essential to ensure symptom resolution. Plastic surgeons should remain vigilant and avoid premature diagnostic closure, recognizing that postoperative abdominal pain is not always attributable to the abdominal wall or operative field.

Ultimately, these cases highlight the importance of maintaining a broad differential diagnosis in the postoperative evaluation of abdominoplasty patients. Prompt recognition of intra-abdominal causes of pain and early escalation of care can prevent delays in diagnosis, reduce complications, and improve patient outcomes.
